# Facile synthesis of copper nitroprusside chitosan nanocomposite and its catalytic reduction of environmentally hazardous azodyes

**DOI:** 10.1186/s13065-024-01224-0

**Published:** 2024-07-02

**Authors:** Dina A. El Mously, Amr M. Mahmoud, Moustafa Ali Khallaf, Howida S. Mandour, Hany A. Batakoushy

**Affiliations:** 1 Pharmaceutical Analytical Chemistry Department, Faculty of Pharmacy, Cairo University, Cairo, 11562 Egypt; 2 Department of Chemistry, School of Pharmacy, Newgiza University (NGU), New Giza, Km 22 Cairo-Alex Road, Cairo, Egypt; 3 Pharmaceutical Chemistry Department, Faculty of Pharmacy, Cairo University, Kasr El-Aini Street, Cairo, 11562 Egypt; 4 Physical Chemistry Department, National Research Centre, Giza, 12622 Egypt; 5 Pharmaceutical Analytical Chemistry Department, Faculty of Pharmacy, Menoufia University, Shebin Elkom, Egypt

**Keywords:** Azo dyes, Methyl orange degradation, Wastewater treatment, Catalytic reduction, Nanocatalyst, Cu Nitroprusside, Chitosan

## Abstract

One of the biggest issues affecting the entire world currently is water contamination caused by textile industries’ incapacity to properly dispose their wastewater. The presence of toxic textile dyes in the aquatic environment has attracted significant research interest due to their high environmental stability and their negative effects on human health and ecosystems. Therefore, it is crucial to convert the hazardous dyes such as methyl orange (MO) azo dye into environmentally safe products. In this context, we describe the use of Copper Nitroprusside Chitosan (Cu/SNP/Cts) nanocomposite as a nanocatalyst for the chemical reduction of azodyes by sodium borohydride (NaBH_4_). The Cu/SNP/Cts was readily obtained by chemical coprecipitation in a stoichiometric manner. The X-ray diffraction (XRD), X-ray photoelectron spectroscopy (XPS), and Fourier transform infrared (FT-IR) spectroscopy were applied to investigate chemical, phase, composition, and molecular interactions. Additionally, Scanning electron microscope (SEM) was used to examine the nanomaterial's microstructure. UV–vis spectroscopy was utilized for studying the Cu Nitroprusside Chitosan's catalytic activity for the reduction of azodye. The Cu/SNP/Cts nanocomposite demonstrated outstanding performance with total reduction time 160 s and pseudo-first order constant of 0.0188 s^−1^. Additionally, the stability and reusability study demonstrated exceptional reusability up to 5 cycles with minimal activity loss. The developed Cu/SNP/Cts nanocomposite act as efficient nanocatalysts for the reduction of harmful Methyl orange azodye.

## Introduction

The environment and human health have been significantly impacted over the past few decades by the rapid and uncontrollable growth of the world’s population as well as technological advancements in the industrial and agricultural sectors [1, 2]. Textile industry is one of the largest contributors to environmental pollution of all the sources due to the use of various toxic dyes [3]. About 50,000 tons of dyes are released into the environment each year as a result of textile wastewater discharge, which contributes 20% of water pollution [4].

The effluents from the textile industries comprise a vast number of intermediate substances because of the presence of numerous unit operations [5]. Furthermore, oxidation and hydrolysis of commercial dyes with azo functional groups can produce toxic and mutagenic byproducts, which contributes to environmental and economic risks. These wastewaters pose a major threat to the environment, threaten sustainable development, and require the development of innovative treatment technologies for the removal and degradation of the toxic dyes [6].

Nevertheless, it is challenging to degrade most of these organic dyes using an efficient and compelling strategy due to their reasonable stability [7]. Azodye degradation from environmental wastewater has been demonstrated using various strategies such as adsorption [8], ion exchange microbial degradation [9], photocatalytic degradation [10], electrocatalytic reduction [11]. Nevertheless, the forementioned strategies have various limitations such as the costly nature of some adsorbents and membranes (adsorption, ion exchange), inadequate degradation rate (microbial degradation) and the possibility of corrosion of electrodes (electrocatalytic reduction).

Despite the limitations with aforementioned methods, it was proven that reduction of azodye into harmless products utilizing Sodium Borohydride (NaBH_4_) could be efficiently inducted in the presence of a convenient catalyst. This approach is distinguished by its ease of use, time saving, efficient conversion rates and lack of sludge [12–19].

Reduction of azodyes including methylorange (MO) using NaBH_4_ needs a catalytic system to overcome the large kinetic barrier which makes the reaction challenging. Consequently, catalysts facilitate the electron transition between the BH_4_^−^ as an electron donor and MO as an electron acceptor and hence making the reaction favourable [16, 20]. Therefore, it is crucial to produce an affordable, highly stable catalyst for the reduction of azodyes.

Over the last decades, there has been a lot of interest in the use of nanocatalysts in the reduction of azodyes such as methyl orange [13, 19]. Nanocatalysts have been found to be efficient for the reduction of methyl orange due to their high surface area for electron transfer than conventional powdered catalysts. Various nanocatalysts, including metal nanoparticles, have been reported in the literature for catalytic reduction of MO such as Cu NPs [21, 22], Au NPs [23], Fe_3_O_4_@-Ag NPs [24]. Despite their effective catalytic function, the need for more affordable and simpler nanocatalysts has prompted more research in this field.

Pentacyanonitrosylferrate(II) known as nitroprusside ion [Fe(CN)_5_NO]^2−^ is a coordination complex capable of forming various substituted metal nitrosyl pentacyanides imposing various 3D structures with diverse properties and applications that vary according to the type of transition metal complexing with it [25]. With an immense similarity between nitroprusside and Prussian blue (ferric ferrocyanide (Fe^3+^)_4_[(Fe^2+^)(CN)_6_]_3_) in terms of chemical composition, electronic structure, redox properties, and electrocatalytic activities, it can be assumed that nitroprusside transition metal complexes might possess similar catalytic activity as Prussian blue transition metal analogues that have been utilized for decomposition of environmental pollutants [6, 25].

Chitosan, a biopolymer sourced from chitin, functions as a stabilizing and size-controllable agent to enhance the dispersion and stability of nanoparticles, thereby complementing their catalytic activity [26, 27]. This combination enhances the overall efficiency of the catalytic system, providing a sustained catalytic activity over multiple reaction cycles. Chitosan provides more functional groups that can take part in the reduction process, which further enhances the catalytic system's reactivity [28]. The azodyes may interact with the amino and hydroxyl groups in chitosan facilitating their adsorption onto the catalytic surface and advocating better contact between the dye molecules and nanoparticles active sites. Furthermore, the NP-Chitosan composite shows enhanced stability across a wide pH range, which makes it adaptable for use in various wastewater treatment conditions. Therefore, the NP-Chitosan enhances the reaction kinetics, and the addition of chitosan provides an eco-friendly and biocompatible matrix for the nanoparticle entrapment, providing a sustainable approach towards water quality improvement [28].

Based on previous knowledge of Prussian blue analogues catalytic activity and chitosan capability to stabilize nanoparticles and enhance their catalytic activity, the current study describes the synthesis of Cu Nitroprusside Chitosan (Cu/SNP/Cts) nanocomposite for the first time and their potential use as nanocatalysts for the catalytic reduction of MO utilizing NaBH_4_ as a reducing agent.

## Experimental

### Chemicals and reagents

Sodium borohydride (NaBH_4_) 98% purity, Methyl orange ≥ 98% purity, Chitosan ≥ 99% purity, Copper sulphate ≥ 99% and Sodium nitroprusside were purchased from Sigma Aldrich (Germany). Ultrapure water was attained from “Aquatron” Automatic Water Still A4000, Bibby Sterillin Ltd., (Staffordshire, UK).

### Synthesis of copper nitroprusside/chitosan nanocomposite

The Cu/SNP/Cts nanocomposite was synthesized according to the reported method [29]. In a 100 mL solution of HCl, (0.5 mol L^−1^), 300 mg of Chitosan (200 k) powder was dissolved and stirred at ambient temperature for one hour, then 20 mL solution of 1 mM sodium nitroprusside was added to 80 mL chitosan solution and left on stirrer for 30 min. After that, 20 mL solution of 1 mM copper sulfate was added in a dropwise manner over 30 min under stirring where solution turned bluish green over time. Then, 200 mL acetone was mixed with the solution after 1 h and centrifuged at 4000 rpm for 15 min to collect and wash the formed nanoparticles at which washing with acetone and centrifugation were repeated several times. The formed Cu/SNP/Cts nanoparticles were finally washed with methanol and left to dry out.

### Characterization of copper nitroprusside/chitosan nanocomposite

The chemical composition of Cu/SNP/Cts was investigated utilizing Thermo Fisher Scientific ESCALAB (USA) for X-ray photoelectron spectroscopy (XPS) analysis. Fourier transform infrared (FTIR) spectra were attained utilizing a Vertex 80V FTIR spectrometer (Bruker, Germany) for function group analysis. The Cu/SNP/Cts complex’s crystal structure was examined using X-ray diffraction (XRD) (Model: Rigaku Smart Lab.). An electron microscope (SEM; Japan Electro Company) coupled to energy-dispersive X-ray analysis for elemental mapping (EDX) was utilized to study the surface morphology of the synthesized Cu/SNP/Cts to identify the primary elemental composition and purity of the nanoparticles. A Shimadzu UV–Visible double-beam spectrophotometer, model 1601 PC (Japan), was used to monitor the reduction reaction’s completion.

### Study of the catalytic activity of Cu/SNP/Cts towards reduction of MO

The reduction of MO was performed in an aqueous medium utilizing sodium borohydride as a reducing agent and Cu/SNP/Cts as nanocatalyst. The progression of the reduction process was monitored by UV–vis spectrophotometry. In this study, 2.5 mL of BR buffer pH 9.0 was added in a quartz cuvette along with 100 μL of an aqueous solution of MO (C = 3 mM), 200 μL of an aqueous solution of NaBH_4_ (C=300 mM), and 5 μL of an aqueous suspension of Cu/SNP/Cts (C = 1000 μg mL^−1^). At consistent intervals, the UV–vis spectra have been recorded over the range of 200–550 nm. The absorbance of MO at various time intervals at 464 nm was measured to track the reaction’s progression at room temperature. Blank experiments were furthermore done by repeating the same procedures but with using NaBH_4_ alone without a catalyst.

## Results and discussion

### Structural, composition, and molecular interaction analysis

#### Structural analysis and surface compositions

##### Transmission electron microscopic analysis of the composite Cu/SNP/Cts

**Fig. 1:**
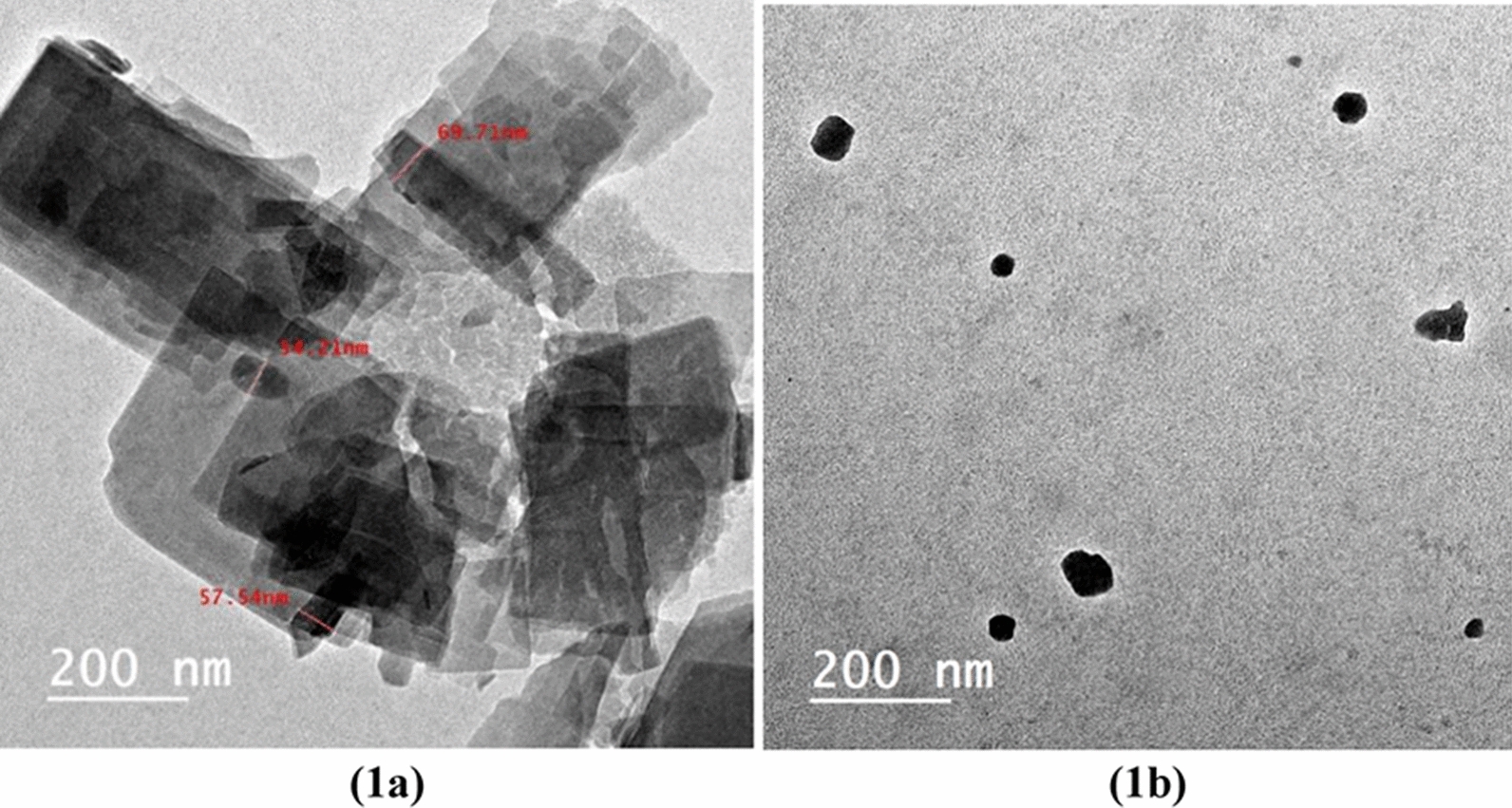
TEM image of the synthesized nanocomposite (Cu/SNP/Cts)

Transmission electron microscopic (TEM), technique is a useful and good tool to understand and determine the size, shape, structure, dispersion, uniformity of material, and crystal phase. It can be observed from Fig. 1a, that; the core shape of the composite (Cu/SNP/Cts) appeared as irregular thin sheets containing particles in the range of nanoscale 57–69 nm; this result indicates that, the synthesized sample can be identified as a nanocomposite (Cu/SNP/Cts). In addition, from Fig. 1b, it can be noticed that these nanoparticles are in a spherical shape. Based on the previous studies [30, 31], the copper nitroprusside nanoparticles usually appear in this shape.

##### XRD analysis (X-ray diffraction)

**Fig. 2:**
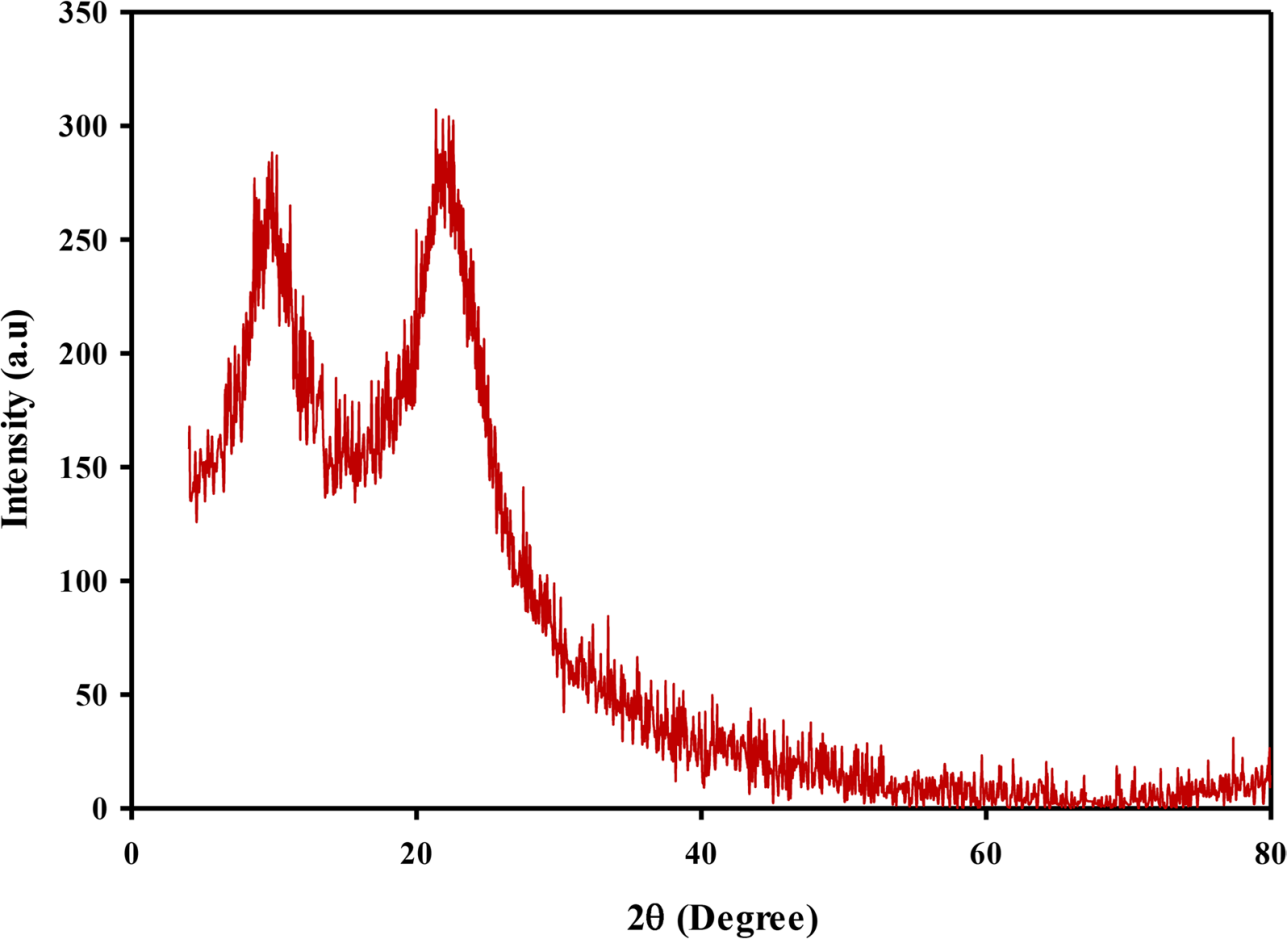
XRD spectrum of pure chitosan (Cts)

**Fig. 3:**
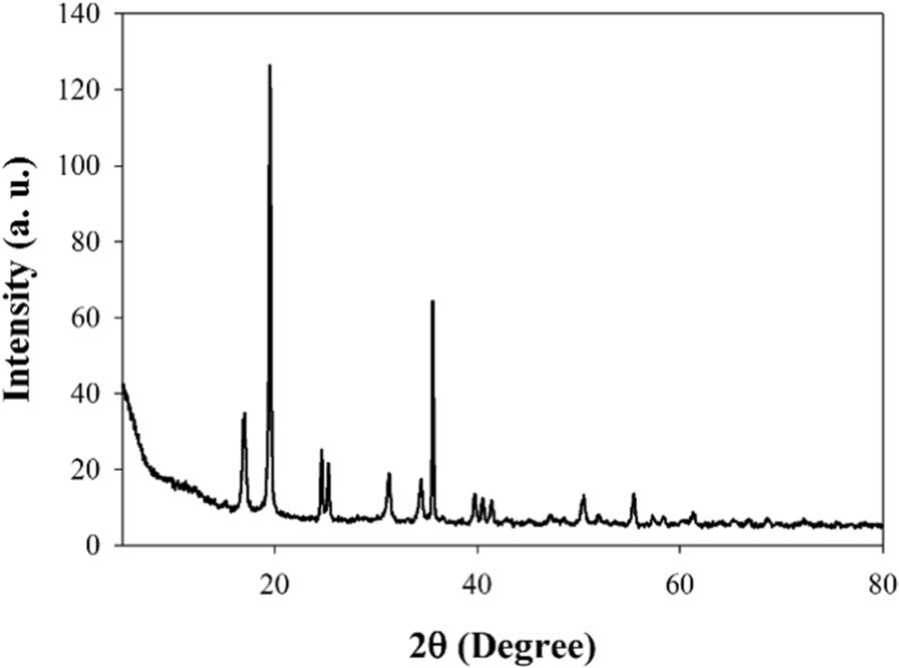
XRD spectrum of the synthesized nanocomposite (Cu/SNP/Cts)

XRD is considered a faster analytical technique used for phase identification of crystalline materials and can provide information on unit cell dimensions. The spectrum of x-ray diffraction for the chitosan (Cts), is presented in Fig. 2. As, it can be seen from Fig. 2, that, the XRD manner of (Cts), displayed two broad diffraction peaks at 2θ = 9.78° and 21.66°; the existence of these two diffraction angle had been related to the crystallinity of the chitosan [32]. The XRD type of the prepared nanocomposite (Cu/SNP/Cts), is shown in Fig. 3. It can be noticed from Fig. 3; the presence of the diffraction peaks at 2θ = 36.40°, 43.47°, 50.91° and 75.21°, which are related to the crystal plane (111), (111), (200) and (220), respectively, emphasizes the formation of the cubic lattice of copper nanoparticles Cu(NPs), owing to (JCPDS No. 040836), [33]. Also, according to previous studies; the diffraction peak at 19.55° was assigned to the existence of chitosan (Cts) in the medium [34]. It can be observed from Fig. 3, that the diffraction peak of the chitosan (Cts) recorded the highest peak intensity among the others. This observation confirms the interaction between the nanoparticles of the prepared nanocomposite and the stabilizing medium (Cts) [35]. Also, the spectrum of Fig. 3, shows the diffraction peaks at 2θ = 17.61°,24.63°,35.62° and 39.74°, corresponding to the crystal plane (200), (220), (222), and (400), respectively, assures the formation of cubic structure of Copper Nitroprusside nanoparticles SNP(NPs), [25, 36]. This result was in matching with the reference data of PB (ICDD PDF2 01-073-0687). Therefore, through this result; it can be deducted that, the synthesized nano-composite is (Cu/SNP/Cts). The crystal size was measured using Debye-Scherer Eq. (1):

1 $${\text{D}}\, = \,{\text{K}}\lambda /\beta {\text{ cos }}\theta$$

where D is the crystal size of the nanoparticle, λ is 0.15406 nm (wavelength of X-ray), K is the Scherer’s constant or shape factor with a value of 0.9, θ is the diffraction angle, and β1/2 is the full width half maxima FWHM [37]. It was found that the calculated particle size was in the range of 57–82 nm; this result was in agreement with the result investigated from the analytical technique transmission electron microscopic (TEM), which confirms that the synthesized composite (Cu/SNP/Cts) is in the nanoscale range.

##### FT-IR spectra

**Fig. 4:**
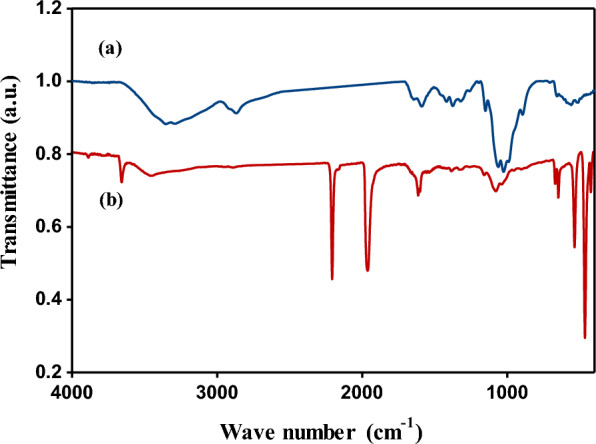
FT-IR spectra of **a** pure chitosan and **b** the prepared nano-composite (Cu/SNP/Cts)

Fourier-transform infrared spectroscopy (FT-IR), of the synthesized nano-composite (Cu/SNP/Cts), and pure chitosan (Cts), was measured and shown in Fig. 4. As, can be noticed from Fig. 4b, that, there are new two bands; one of these bands was sharp and appeared in a weak shape at 3651.26 cm^−1^, and the other band was sharp and appeared in strong shape at 458.57 cm^−1^.

The appearance of these two new bands at (3651.26 and 458.57 cm^−1^), referred to the reaction that occurred between the amino and hydroxyl groups of the chitosan (Cts), and the surface of the Cu nitroprusside nanoparticles [35], or by another words; the Cu nitroprusside NPs had been incorporated into the polymer (Cts).

In addition, it can be seen from Fig. 4b, the appearance of stretching and a broad band in a weak shape at 3454.12 cm^−1^, which corresponds to the overlap of the hydroxyl and amino groups [38], while the amino group (NH_2_), in Fig. 4a, for pure chitosan (Cts), appeared as a broad band at the wave numbers 3229 and 3238 cm^−1^. According to the literature, the cyanide group C≡N appears in the range of the wave number 2220–2260 cm^−1^, from Fig. 4b, it can be observed that, the peak of cyanide group appeared as a strong band in a sharp shape at 2200.64 cm^−1^; decreased than its value of the wave number of literature by about 19 cm^−1^. This result indicates that, the shifting of this peak was attributed to its coordination with Cu in the Cu nitroprusside nanoparticles [39].

As well as, from Fig. 4b, the appearance of a strong band in medium shape at 1956.29 cm^−1^, was related to (NO), group; the presence of these two absorption bands at 2200.64 and 1956.29 cm^−1^, confirmed the formation of pentacyanonitrosylferrate or in other words the formation of a binuclear complex of iron and copper as it was reported previously [40, 41].

Also, from Fig. 4b, the two weak peaks appeared at 1607.62 and 1595.29 cm^−1^, corresponded to the vibration band C=O with shifting decreased by about 26 cm^−1^, in comparison with that of Fig. 4a, of pure chitosan which appeared at 1633 cm^−1^, and the peak of –NH_2_ bending with shifting raised by about 21 cm^−1^, in comparison with that of Fig. 4a, which appeared at 1574 cm^−1^ [31].

All the other absorption bands in Fig. 4b, which appeared at (1379, 1312, 1152 and 1072 cm^−1^), had been changed with shifting decreased or increased than that of Fig. 1a for pure (Cts), which appeared at (1382, 1331, 1162 and 1067cm^−1^), respectively. The two weak peaks at 1379 and 1312 cm^−1^ had been related to C–H bending, and the two broad weak peaks at 1152 and 1072 cm^−1^ belonged to C–O–C group owing to skeletal stretching [38]. The strong medium peak at 530 cm^−1^ in Fig. 1b, was attributed to the metal–carbon interaction; in other words, the linke between Cu and C [30].

##### X-ray photoelectron spectroscopy data analysis

**Fig. 5:**
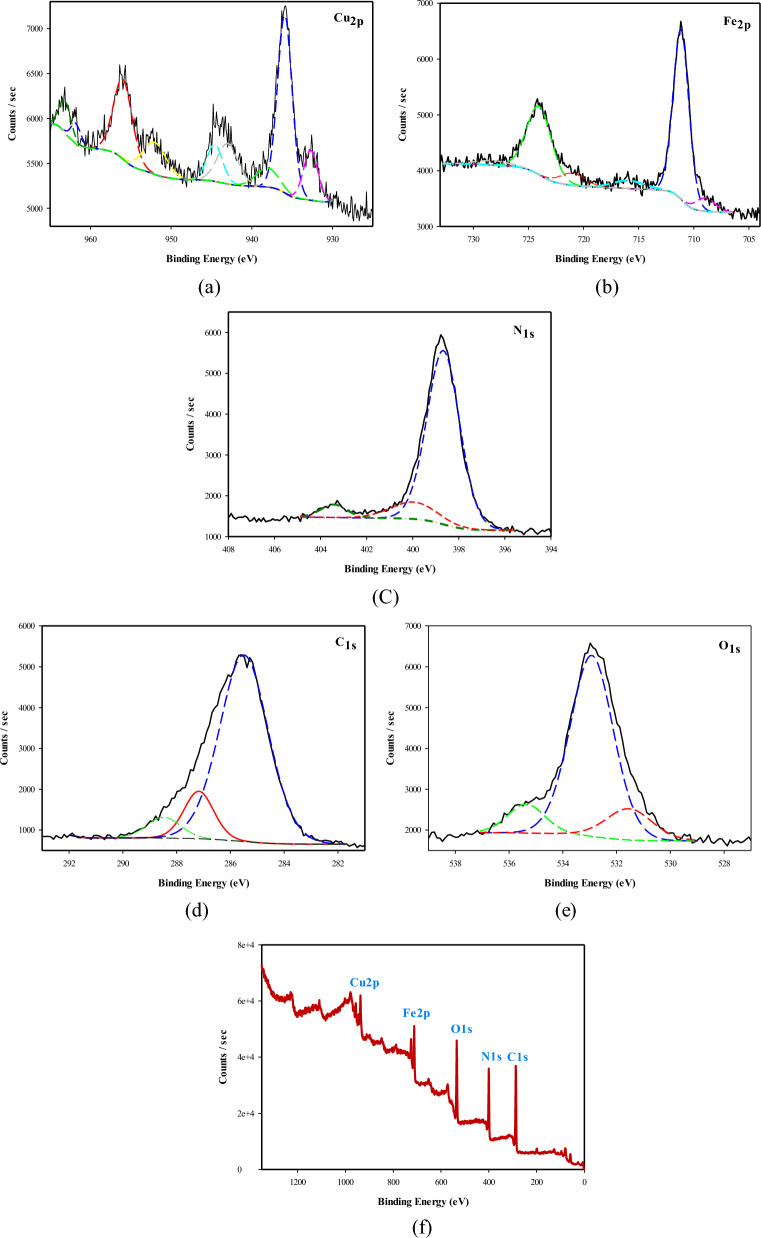
XPS spectra of the synthesized nanocomposite (Cu/SNP/Cts)

XPS is a useful technique which used to analyse the elements constituting of the sample surface (composition, chemical bonding and kinetic energy of the emitted photoelectrons). Based on Fig. 5a, which shows different oxidation states of copper; it can be seen four peaks at binding energy 932.8, 936.2, 952.34 and 956.4 eV; the peak at 932.8 eV was attributed to Cu 2p_3/2_ which confirms the presence of Cu (0) and Cu (I), where the peak around 936 eV, can be assigned to Cu (II) [42]. The peak at 952.34 eV, was related to Cu 2p_1/2_ which revels the existence of Cu (0) and Cu (I), where the peak at the binding energy 956.4 eV, belonged to Cu 2p_5/2_ pointed to the presence of Cu (II). The Fe2p spectrum in Fig. 5b, shows four peaks [43–45] at binding energies of  708.837, 711.192, 720.919, and 724.178 eV. The two high intensity peaks with binding energy 711.192 and 724.178 eV are confirming the presence of Fe _2p1/2_ and Fe _2p3/2_ with Fe^2+^ low spin states, respectively [43–45]. Also, the two binding energy 708.837 and 720.919 eV, are assigned to the high-spin Fe^2+^ state of Fe _2p3/2_ and Fe _2p1/2_, respectively [43–45]. In addition, from Fig. 5c, three peaks at binding energy 398.726, 399.95 and 403.276 eV, can be observed, which corresponded to the presence of nitrogen atom at 398.726 eV, and the other two broad peaks are related to the existence of C=N and C≡N groups with sp^3^ and sp^2^ hybridization, respectively [46]. As well, Fig. 5d, illustrated the appearance of three broad peaks at binding energy 285.5, 287.1 and 288.7 eV, which are related to the presence of C=N, C≡N groups with sp^2^ and sp^3^ hybridization, respectively and the third broad peak at binding energy 288.7 eV, was attributed to O–C=O group [46]. Figure 5f, shows three broad peaks at binding energy 531.3, 532.9 and 535.4 eV, corresponding to formation of Fe (OH)_3_, and presence of C–O–C group respectively, while the high binding energy 535.4 eV, was assigned to existence of ether group R-O-R [47].

##### Surface morphology and constituent of the nano-composite (Cu/SNP/ (Cts)

**Fig. 6:**
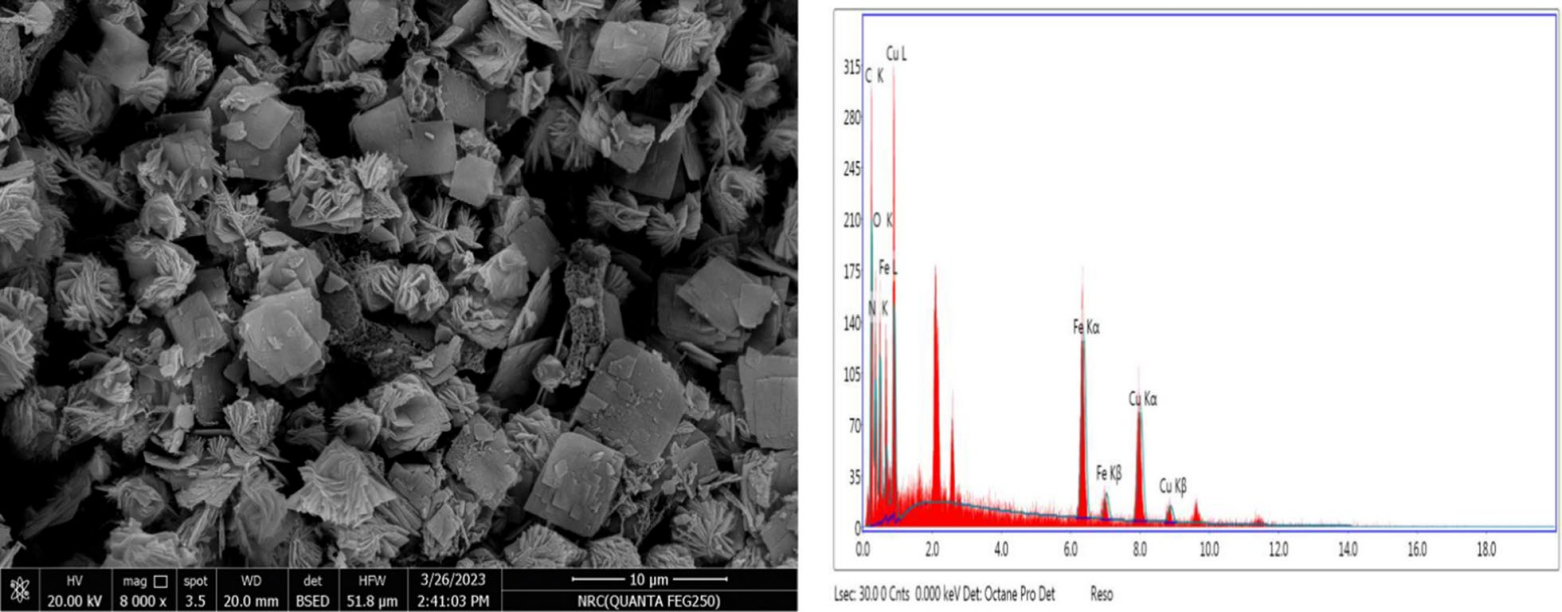
SEM image and EDX analysis of the synthesized nanocomposite (Cu/SNP/Cts)

It was clear from the image of scanning electron microscopy analysis (SEM), that, the nanoparticles of the nanocomposite (Cu/SNP/Cts), had been grown  in the shape of nano-sheet with higher agglomeration resulted in formation of large flocks of these nanoparticles. The large surface area and also high ability of copper nanoparticles to bond may be the reason of the agglomeration of the nanoparticles [31]. The constituent composition of the synthesized nanocomposite (Cu/SNP/Cts), was investigated by energy dispersive X-ray spectrometer (EDX), analysis, which confirms the presence of all the basic elements to form the nanocomposite (Cu/SNP/Cts), such as, copper, iron and the elements; carbon, oxygen, and nitrogen, as reported in Table 1 and shown in Fig. 6.

**Table 1:** The compositions of the main elements of the synthesized Cu/SNP/Cts

Element	Weight %	Atomic %
C	24.37	36.2
O	16.79	18.72
N	28.26	35.99
Fe	13.18	4.21
Cu	17.4	4.89

### Catalytic reduction of methyl orange using Cu/SNP/Cts nanocomposite

Azo dyes like methyl orange (MO) are regarded as one of the most basic pollutants in wastewater and natural water bodies.MO pose significant environmental risks due to its high potential for being teratogenic, and carcinogenic and toxic azodye. Hence, it is vital to transform the hazardous MO azo dye into less harmful substances, such as 4-aminobenzenesulfonic acid and N,N dimethylbenzene 1,4 diamine (DMBD). 4-aminobenzenesulfonic acid, also known as sulfanilic acid, is an indirect FDA-approved food additive.

**Fig. 7:**
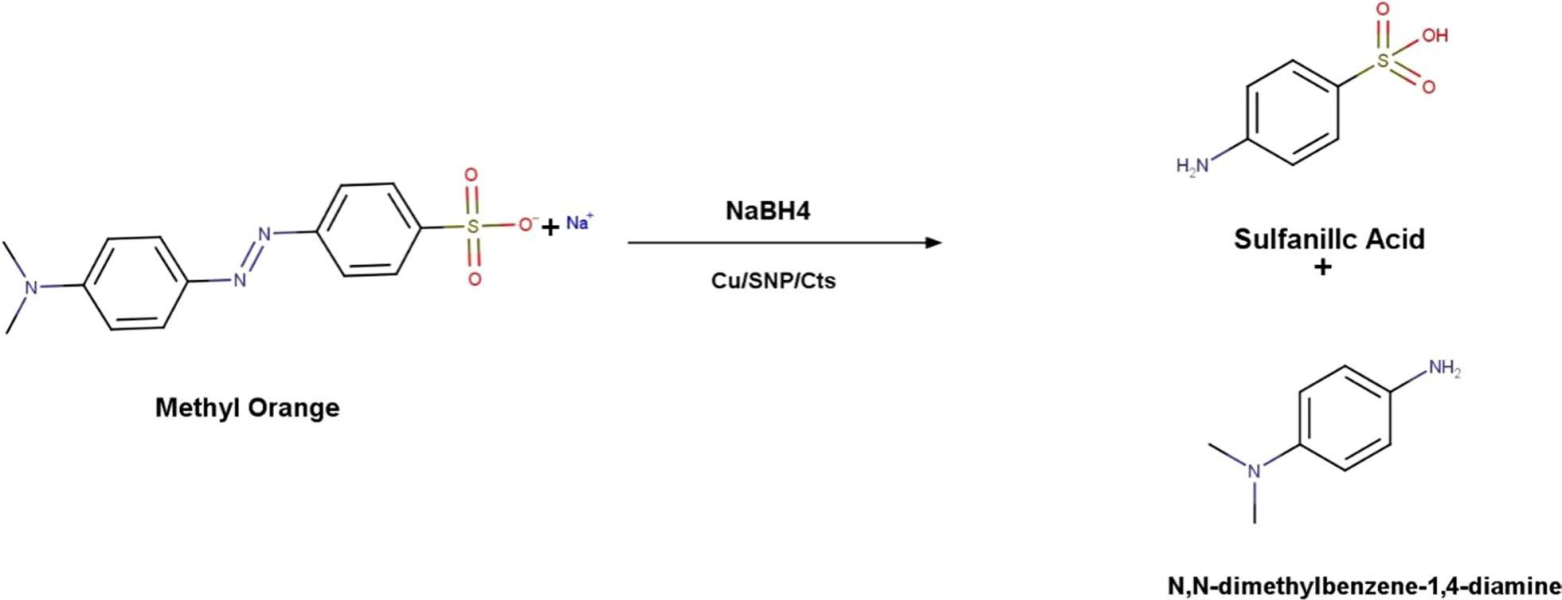
Reduction of MO by NaBH_4_ utilizing Cu/SNP/Cts as nanocatalyst

**Fig. 8:**
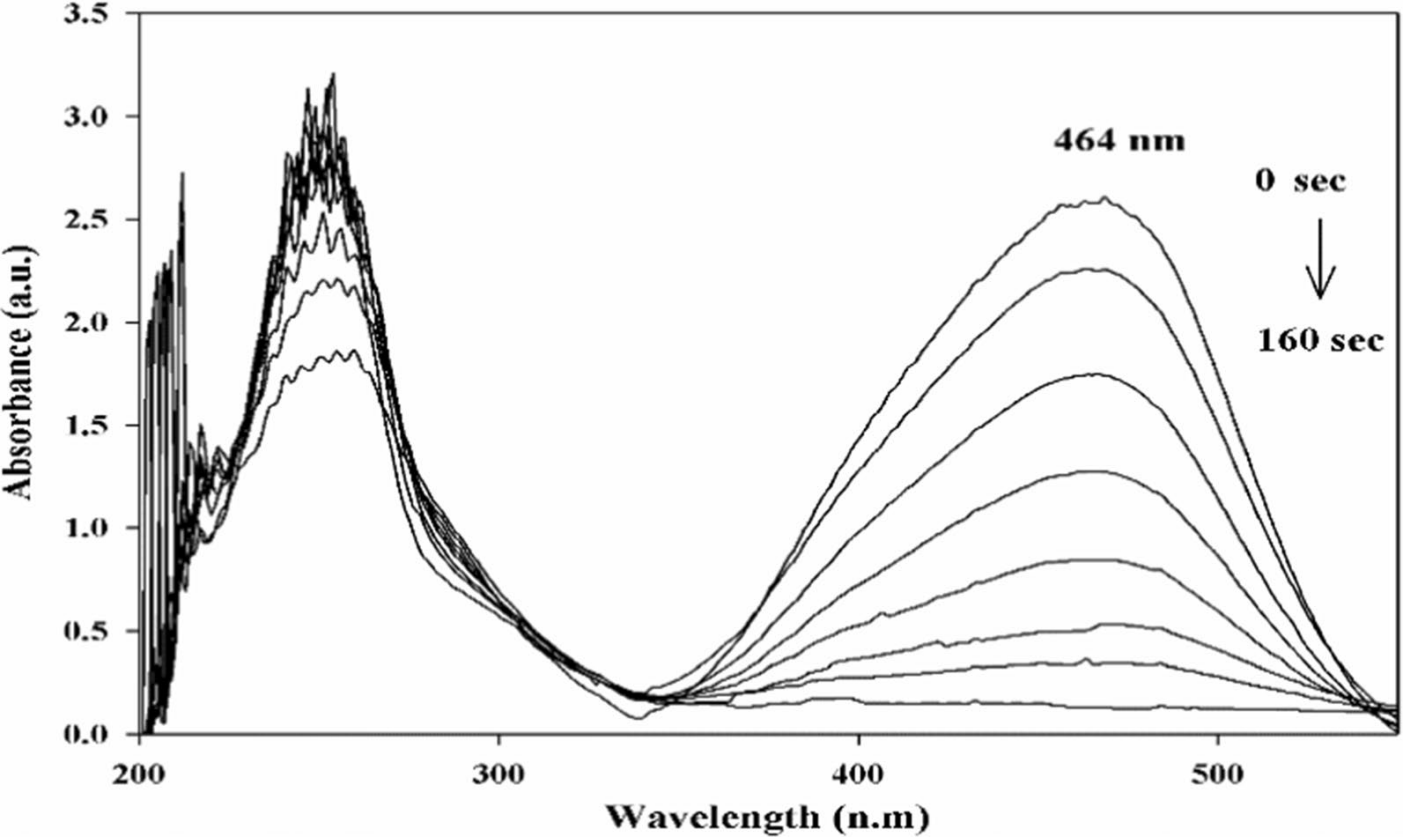
UV–vis spectra for the reduction of Methyl orange compound by Cu/SNP/Cts at distinct time intervals

**Fig. 9:**
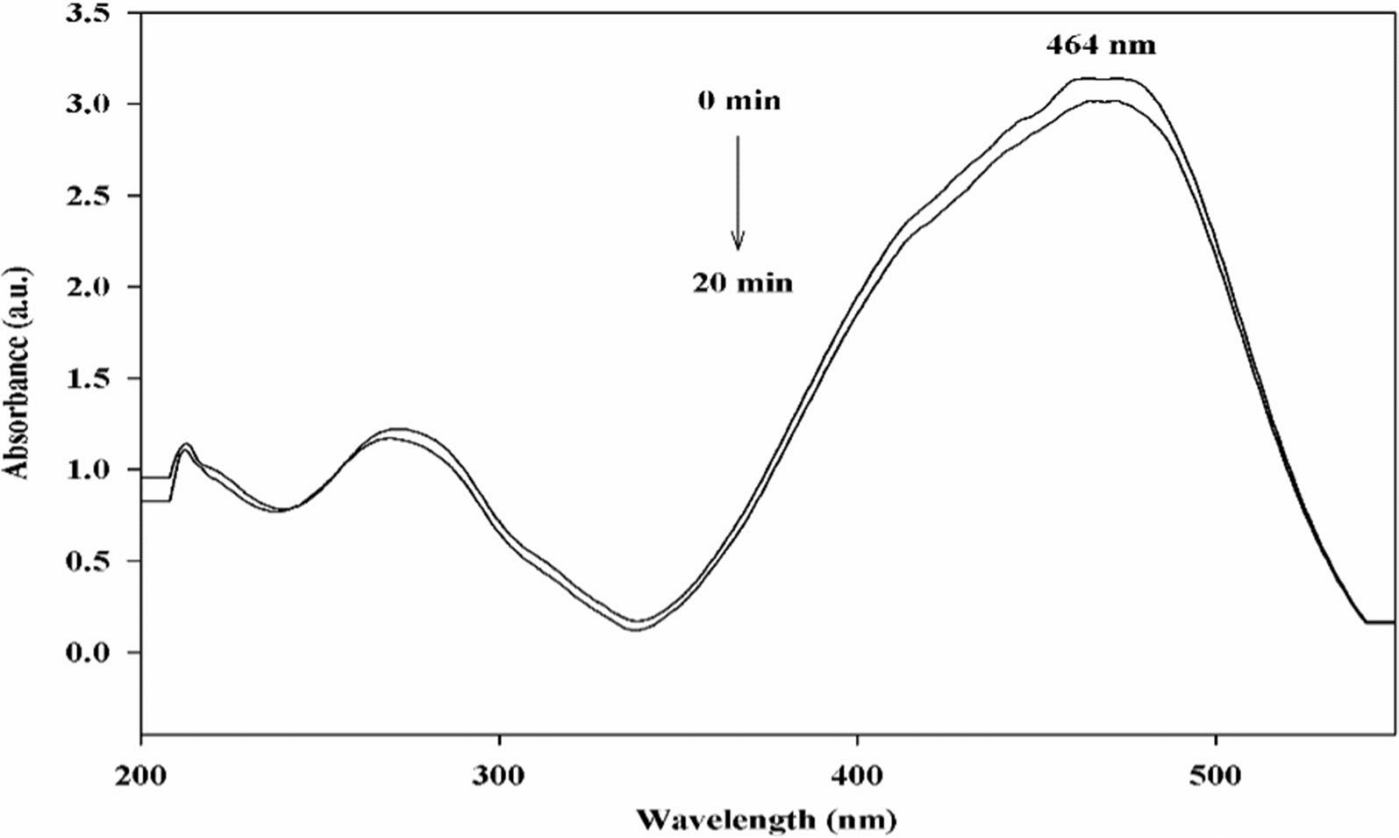
UV–visible spectra for Methyl orange reduction without using a nanocatalyst

MO was catalytically reduced utilizing NaBH_4_ and Cu/SNP/Cts nanocatalyst (Fig. 7). Using UV–VIS spectrophotometry, the reduction reaction's process was tracked over time. The azo bond and the dimethylamino electron-donating groups are responsible for the absorbance at 464 nm in the MO spectrum. The intensity of the MO peak at 464 nm was instantly decreased after adding the Cu/SNP/Cts nanocatalyst, and it vanished entirely after 160 s (Fig. 8). The formation of hydrazine derivatives was indicated by the appearance of a new peak at 250 nm. The MO's yellow colour fully disappeared after 160 s, indicating that it had been completely diminished. Additionally, the reduction of MO was explored without utilizing the Cu/SNP/Cts nanocatalyst. A very small reduction in the peak intensity of MO was observed at 464 nm, Fig. 9.

NaBH_4_ can reduce MO in a thermodynamically feasible manner. However, the reaction is kinetically challenging due to the high kinetic barrier between MO, an electron acceptor, and NaBH_4_, an electron donor.

**Fig. 10:**
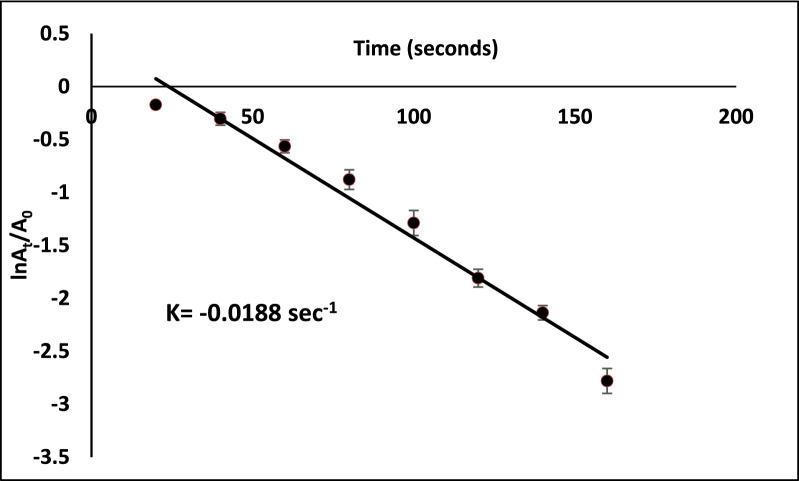
Kinetics plot of ln(A_t_/A_o_) against time for reduction of Methyl orange utilizing Cu/SNP/Cts as nanocatalyst

A pseudo-first-order kinetic model was implemented to compute the rate constant for MO reduction because of the excessive concentration of NaBH_4_ compared to that of MO. It was presumed that the rate of reaction relied only on the concentration of MO as the concentration of NaBH_4_ remains constant throughout the dye reduction reaction, in accordance with the Langmuire-Hinshelwood model [48, 49]. The equation of pseudo-first-order kinetic is described as ln (*C*_*t*_/*C*_o_) =  − *kt*. It can also be described as ln (*A*_*t*_/*A*_o_) =  − *kt* since they are equal at 464 nm. Hence, ln (A_t_/A_o_) versus the reduction time was plotted in Fig. 10 to show the progression of MO reduction in the presence of Cu/SNP/Cts nanocatalyst. The slope of the plot was utilized to calculate the apparent rate constant (k), which turned out to be 0.0188 s^−1^.

### Effect of Catalyst Dose and Medium pH on Catalytic Activity of Cu/SNP/Cts Nanocatalyst

Under various pH conditions (acidic; pH 5.0, neutral; pH 7.0, and basic; pH 9.0), the catalytic efficiency of Cu/SNP/Cts nanocatalyst towards MO reduction by NaBH_4_ was evaluated. In all experiments, the least possible concentration of nanocatalyst was maintained (5 µg). The alkaline condition (pH 9.0) resulted in the fastest catalytic reduction where the computed k was found to be 0.0188 s^–1^ with a total reduction time 160 s. The rate decreased with decreasing pH, with k values of 0.007 s^–1^ at pH 7.0 and 0.002 s^–1^ at pH 5.0, respectively. At pH 7.0, 69.9% of MO was reduced after 160 s, but at pH 5.0, only 47.9% of MO (Table 2).

**Table 2:** Comparison between the catalytic efficiency of Cu/SNP/Cts nanocomposite in different pH in terms of percentage MO reduction in 160 s and reaction rate *k*

pH	*k*	% MO reduction in 160 s
5.0	0.002 s^−1^	47.9
7.0	0.007 s^−1^	69.9
9.0	0.0188 s^−1^	100

One of the most widely used water-soluble azo dyes, MO is usually dumped in industrial wastewater [50] and is widely used in the textile and paper industries. These industries frequently produce effluent with an alkaline pH [51, 52]. The strong catalytic activity of the suggested nanocatalyst in alkaline pH is therefore of great benefit to remove dangerous MO azo dye in alkaline industrial wastewater, according to the results. The alkaline pH of textile effluent, which affects the growth and metabolism of microbial cells, frequently limits the biological treatment of the waste [53].

### Reusability of the proposed Cu/SNP/Cts nanocatalyst

**Fig. 11:**
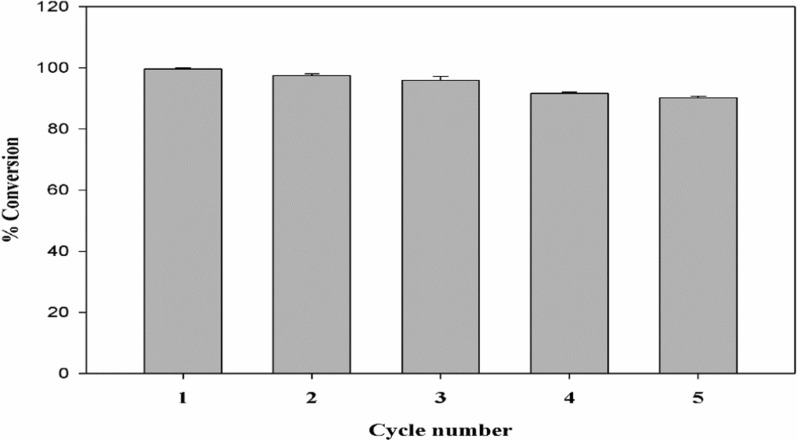
Rate of Methyl orange conversion using Cu/SNP/Cts as a nanocatalyst following five distinct cycles

The nano-catalyst’s efficiency, recyclability, and usability are crucial aspects for practical applications in the degradation process of textile dyes. Economic value can be found by a stable catalyst that can be used repeatedly without losing its effectiveness because it lowers the process's cost. Recycling studies were therefore carried out at the observed conditions to evaluate the recyclability and stability of Cu/SNP/Cts as nano-catalysts and its efficiency in between cycles. This was accomplished by adding a fresh batch of Cu/SNP/Cts NPs to the reaction mixture after one catalytic reduction cycle was completed without catalyst regeneration. The Cu/SNP/Cts nanocatalyst was found to demonstrate nearly the same catalytic performance after five catalytic reduction cycles with a very slight increase in the total reduction time. Nevertheless, even in the absence of regeneration, the Cu/SNP/Cts was still able to reduce MO speedily up till 91% after the fifth cycle revealing good catalytic activity and high stability of these nanocatalysts in this reduction process, Fig. 11.

### Evaluation of the proposed catalytic efficiency of Cu/SNP/Cts in relation to other reported catalysts for MO reduction

The newly synthesized PBA Cu/SNP/Cts was evaluated for its catalytic efficiency against other catalysts reported in the literature for the reduction of MO. Table 3 reveals that the suggested nanocatalyst exhibited exceptional performance. A higher rate constant (k) and a notable decrease in the overall time needed for the full reduction of MO were attained with just 5 µg of the recommended nanocatalyst. To sum up, the suggested PBA outperforms other catalysts documented in the literature in terms of catalytic activity.

**Table 3:** Comparison between the catalytic efficiency of the suggested Cu/SNP/Cts and other documented catalysts for MO reduction

Catalyst	NaBH_4_ amount	Catalyst amount	*k* (s^−1^)	Reaction completion time (min)	Refs.
Cu NPs	0.5 mL (0.2 M)	5000 µg	8.6 × 10^–3^	4	[21]
CuAg/ZnO/carbon black-cellulose acetate sheets	0.5 mL (1 M)	0.5 cm^2^	1. 5 × 10^–3^	12	[54]
rGO–Ag/PVA	N/A	50 µg	0.8 × 10^−3^	12	[55]
C@Fe	1 mL (0.5 M)	5000 µg	15.3 × 10^–3^	4	[15]
DLP-AuNPs	? (1 × 10^–3^ M)	100 µg	1.7 × 10^−3^	8	[56]
SDS@Ag–Cu NPs	0.5 mL (0.025 M)	448 µg/mL	6.4 × 10^–3^	21	[57]
Cu/SNP/Cts	0.2 mL (0.3 M)	5 µg	18.8 × 10^–3^	2.66	This work

## Conclusion

**Scheme 1:**
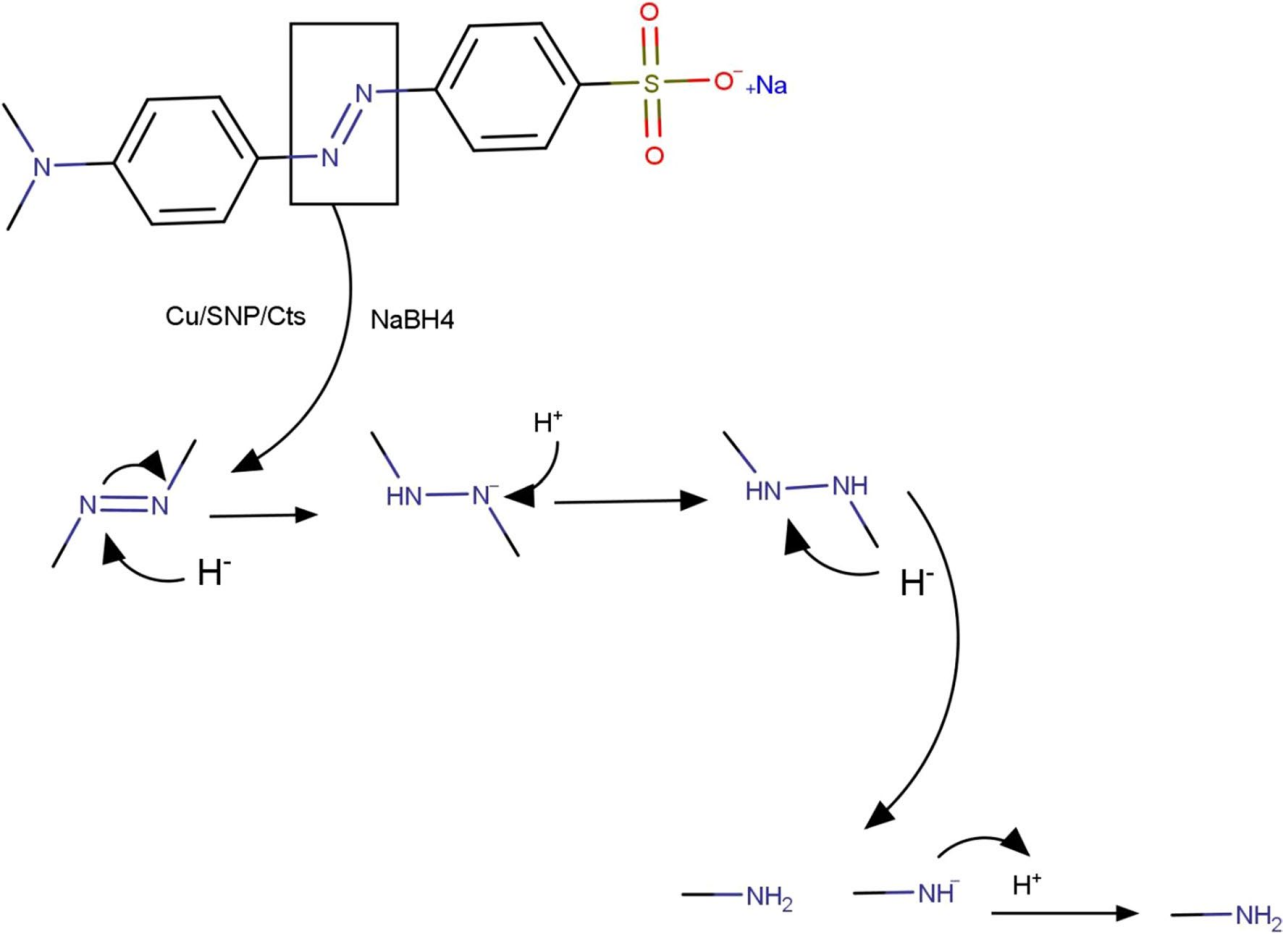
Proposed mechanism of Methyl orange catalytic reduction utilizing NaBH_4_ and Cu/SNP/Cts nanocomposite

The widespread distribution of toxic textile dyes in the aquatic environment has attracted significant research interest because of its detrimental health effects on people as well as its overall environmental impact. In this study, novel Copper Nitroprusside Chitosan nanocomposite was effectively synthesized through chemical coprecipitation to be used as a nanocatalyst for the reduction of widely utilized textile azodye Methyl Orange. The Copper Nitroprusside Chitosan nanocomposite was shown to be a highly intriguing candidate for rapid reduction of the potentially hazardous MO to more benign products. Moreover, the influence of various parameters such as catalyst dose and pH of medium was studied. Following the optimization procedure, it was found that the overall reduction time of MO was 160 s with a calculated rate constant (*k*) equal to 0.0188 s^–1^. Furthermore, the nanocatalyst demonstrated excellent stability and recyclability for 5 repeated cycles with no remarkable decline in catalytic performance. The proposed mechanism of Methyl orange catalytic reduction utilizing NaBH_4_ and Cu/SNP/Cts nanocomposite is presented in Scheme 1. Ultimately, the introduced Copper Nitroprusside Chitosan nanocomposite could be utilized as a nanocatalyst for rapid degradation of the toxic azodyes from environment and consequently could unlock the way to various catalytic and environmental remediation applications.

## Data Availability

All data generated or analyzed during this study are included in this published article.

## Abbreviations

MO: Methyl orange
Cu/SNP/Cts: Cu nitroprusside chitosan
NaBH_4_: Sodium borohydride
XPS: X-ray photoelectron spectroscopy
FTIR: Fourier transform infrared spectrometer
XRD: X-ray diffraction
EDX: Energy-dispersive X-ray
DMBD: N,N dimethylbenzene 1,4 diamine
